# Data on metal-chelating, -immobilisation and biosorption properties by *Gordonia rubripertincta* CWB2 in dependency on rare earth adaptation

**DOI:** 10.1016/j.dib.2020.105739

**Published:** 2020-05-21

**Authors:** Ringo Schwabe, Christoph Helmut Rudi Senges, Julia Elisabeth Bandow, Thomas Heine, Henry Lehmann, Oliver Wiche, Michael Schlömann, Gloria Levicán, Dirk Tischler

**Affiliations:** a Institute of Biosciences, Environmental Microbiology Group, TU Bergakademie Freiberg, Leipziger Straße 29, 09599 Freiberg, Germany; b Institute of Biosciences, Biology and Ecology Group, TU Bergakademie Freiberg, Leipziger Straße 29, 09599 Freiberg, Germany; c Applied Microbiology, Faculty of Biology and Biotechnology, Ruhr University Bochum, Universitätsstraße 150, 44801 Bochum, Germany; d Institute of Informatic, TU Bergakademie Freiberg, Bernhard-von-Cotta Straße 2, 09599 Freiberg, Germany; e Laboratorio de Microbiología Básica y Aplicada, Facultad de Química y Biología, Universidad de Santiago, Chile; f Microbial Biotechnology, Faculty of Biology and Biotechnology, Ruhr-Universität Bochum, Universitätsstraße 150, 44780 Bochum, Germany

**Keywords:** Metallophore, Heavy metals, Rare earth elements, Dissolution, Chelating agent, ACTINOBACTERIA

## Abstract

Recent studies have shown that the metal adaptation of Actinobacteria offers a rich source of metal inducible environmentally relevant bio-compounds and molecules. These interact through biosorption towards the unique cell walls or via metal chelating activity of metallophors with trace elements, heavy metals and even with lanthanides to overcome limitations and toxic concentrations. Herein, the purpose is to investigate the adaptation potential of *Gordonia rubripertincta* CWB2 in dependence of the rare earths and to determine if we can utilize promising metallophore metal affinities for metal separation from aquatic solutions. For details on data interpretation and applicability of siderophores we refer to the related article entitled “Cultivation dependent formation of siderophores by *Gordonia rubripertincta* CWB2” [1].

The respective workflow comprises a metal adaptation method to demonstrate effects on bacterial growth, pH, metallophore production, and metabolic change. All this was evaluated by LC-MS/MS and effects on biosorption of rare earths was verified by ICP-MS. Furthermore, we were able to carry out batch metal adsorption and desorption studies of metallophores entrapped in inorganic polymers of tetramethoxysilane (TMOS) to determine metal chelating capacities and selective enrichment effects from model solutions. The adaptation potential of strain CWB2 at increased erbium and manganese concentrations was verified by increased chelating activity on agar plates, in liquid assays and demonstrated by the successful enrichment of erbium by metallophore-functionalized TMOS-polymers from an aquatic model solution. Furthermore, the number of detected compounds in dependency of rare earths differ in spectral counts and diversity compared to the wild type. Finally, the biosorption of rare earths for the selected adaptation was increased significantly up to 2-fold compared to the wild-type. Overall a holistic approach to metal stress was utilised, integrating a bacterial erbium adaptation, metal chelating, biosorption of lanthanides and immobilization as well as enrichment of metals using metallophore functionalized inorganic TMOS polymers for separation of metals from aquatic model solutions.

Specifications table

**Table:** 

Subject	Applied Microbiology and Biotechnology
Specific subject area	We report on production and nature of siderophores obtained from the Actinobacterium *G. rubripertincta* CWB2. Production was optimized and an application is highlighted.
Type of data	Table Image Graph Figure
How data were acquired	Images of agar plates were taken by camera, XAD extracts were chromatographed by HPLC (Ultimate 3000, Thermo) and FPLC (AKTA Start, GE Healthcare), metal concentrations were measured by mass spectrometry (ICP-MS, Thermo), metabolites in extracts were analysed by LC-MS/MS-ESI (Waters), pH profiles of cultivations were tracked by PreSens pH sensor (PreSens Precision Sensing GmbH). Software and programs: antiSMASH4 (Weber, 2015), Proteowizard (Kessner et al., 2008), GNPS (Wang and et. al, 2016), MetFrag (Ruttkies et al., 2016).
Data format	Raw: Provided via a data repository: LC-MS/MS. Analyzed and Filtered: Provided as EXCEL file.
Parameters for data collection	We investigated different media compositions (ionic strength, phosphate concentration, additional trace elements and vitamins and metal induced stress) to optimize metallophore production. Further, we investigated adsorption and desorption of metals by metallophore functionalized inorganic polymers of tetramethoxysilane (TMOS) from model solutions at pH 4. We analysed pH profiles during siderophore production from pH 5 to pH 9.
Description of data collection	Herein we combine a CAS agar assay with additional metals. A metal adsorption screening of TMOS entrapped siderophore XAD extracts was performed. We investigated pH differences in dependence of rare earths, to relate the siderophore production along the pH. Further, we investigated an adaptation method for manganese and rare earths to get more insights on bacterial metal adaptation potential. The combination of functional CAS assays and quantitative methods (ICP-MS and LC-MS/MS-ESI) gives insights to the metal interaction potential.
Data source location	Institution: TU Bergakademie Freiberg City/Town/Region: Freiberg Country: Germany
Data accessibility	Repository name: GNPS (gnps.ucsd.edu) Data identification numbers: Gordonia rubripertincta CWB2 siderophores: Positive mode: bbd4f668b6224bd381617001be917381 Negative mode: e3009a90a0954791ae9b747e727c6515 Gordonia rubripertincta CWB2 and erbium: Positive mode: 62acd01d08ce4683941473ea55ab0701 Negative mode: f1150f29b2584369a965bab7b94f3764 Repository name: MassIVE (massive.ucsd.edu) Data identification numbers: Gordonia rubripertincta CWB2 siderophores: Positive mode: MSV000084154 Negative mode: MSV000084155 Gordonia rubripertincta CWB2 and erbium: Positive mode: MSV000084608 Negative mode: MSV000084609 Direct URL to data: Gordonia rubripertincta CWB2 siderophores: Positive mode: https://massive.ucsd.edu/ProteoSAFe/dataset.jsp?task=ef5fd7dd5f0046d3b5d7d477e511aa8c ftp://massive.ucsd.edu/MSV000084154
	http://gnps.ucsd.edu/ProteoSAFe/status.jsp?task=bbd4f668b6224bd381617001be917381 Negative mode: https://massive.ucsd.edu/ProteoSAFe/dataset.jsp?task=96c5407032404bccb66789b0f0694d27 ftp://massive.ucsd.edu/MSV000084155 http://gnps.ucsd.edu/ProteoSAFe/status.jsp?task=e3009a90a0954791ae9b747e727c6515 Gordonia rubripertincta CWB2 and erbium: Positive mode: https://massive.ucsd.edu/ProteoSAFe/dataset.jsp?task=acd70f73c4f645ca9f20fa83765d2d9a
	ftp://massive.ucsd.edu/MSV000084608/ https://gnps.ucsd.edu/ProteoSAFe/status.jsp?task=62acd01d08ce4683941473ea55ab0701 Negative mode: https://massive.ucsd.edu/ProteoSAFe/dataset.jsp?task=07ee4fffeb7c4aefae3eae6e013ce5bb ftp://massive.ucsd.edu/MSV000084609/ https://gnps.ucsd.edu/ProteoSAFe/status.jsp?task=f1150f29b2584369a965bab7b94f3764
Related research article	Authors: Ringo Schwabe, Christoph Helmut Rudi Senges, Julia Elisabeth Bandow, Thomas Heine, Henry Lehmann, Oliver Wiche, Michael Schlömann, Gloria Levicán, Dirk Tischler Title: Cultivation dependent formation of siderophores by *Gordonia rubripertincta* CWB2 Journal: Microbiological Research Accepted.

## Value of the data

• The data presented on siderophore production by *Gordonia rubripertincta* CWB2 and its optimization provide a strategy towards novel secondary metabolites. For the first time a rare earth element induced secondary profile was related to secondary metabolites for this genus.
• Natural product scientists and in general microbiologists using GNPS and related tools will use these data, as the published dataset can be used for cross-referencing and identification of recurring molecules.
• An overview on the desferrioxamine diversity allows fingerprinting of desferrioxamine-producing microorganisms. Further, production of certain derivatives enables evaluation of the promiscuity of involved biosynthetic enzymes or can be evaluated with focus on adaptation of the secreted metabolome towards changing environmental conditions.
• The LC-MS/MS-based metabolomics datasets might contain a plethora of new and yet uncharacterized molecules, with interesting biological activities.

## 1 Data Description

In order to identify novel secondary metabolite relevant genes or gene cluster we analysed the whole genome of strain *Gordonia rubripertincta* CWB2 (NCBI Reference Sequence: NZ_CP022580; 5227013 bp) by means of antiSMASH4 [2]. This genome mining resulted in 13 cluster relevant for secondary metabolites, 7 NRPS like, 1 siderophore like, 1 T1PKS, 1 betalactone, arylpolyene, 2 terpene, 1 ectoine and 1 bacteriocin like (see Table 1).

**Table 1:** **antiSMASH4 analysis for *Gordonia rubripertincta* CWB2 chromosome [****2****].** Genome mining for secondary metabolite biosynthesis in *Gordonia rubripertincta* CWB2 chromosome (NCBI Reference Sequence: NZ_CP022580.1; 5227013 bp) allowed to identify the siderophore gene cluster region 2.

Region	Type	From	To	Most similar known cluster	Similarity
Region 1	T1PKS, NRPS-like	193,546	264,747	-	-
Region 2	NRPS, siderophore	611,518	673,907	amychelin	18%
Region 3	NRPS	1,065,001	1,123,788	-	-
Region 4	betalactone, NRPS	2,197,154	2,270,906	streptomycin	7%
Region 5	arylpolyene	2,713,120	2,754,283	primycin	5%
Region 6	terpene	2,922,546	2,941,549	SF2575	6%
Region 7	ectoine	3,276,405	3,286,845	ectoine	75%
Region 8	NRPS	4,069,896	4,180,629	ishigamide	11%
Region 9	NRPS	4,279,788	4,336,675	-	-
Region 10	NRPS	4,481,579	4,525,856	-	-
Region 11	NRPS-like	4,782,290	4,826,162	-	-
Region 12	terpene	4,907,454	4,928,365	carotenoid	33%
Region 13	bacteriocin	4,960,914	4,971,714	amphotericin	11%

**Fig. 1:**
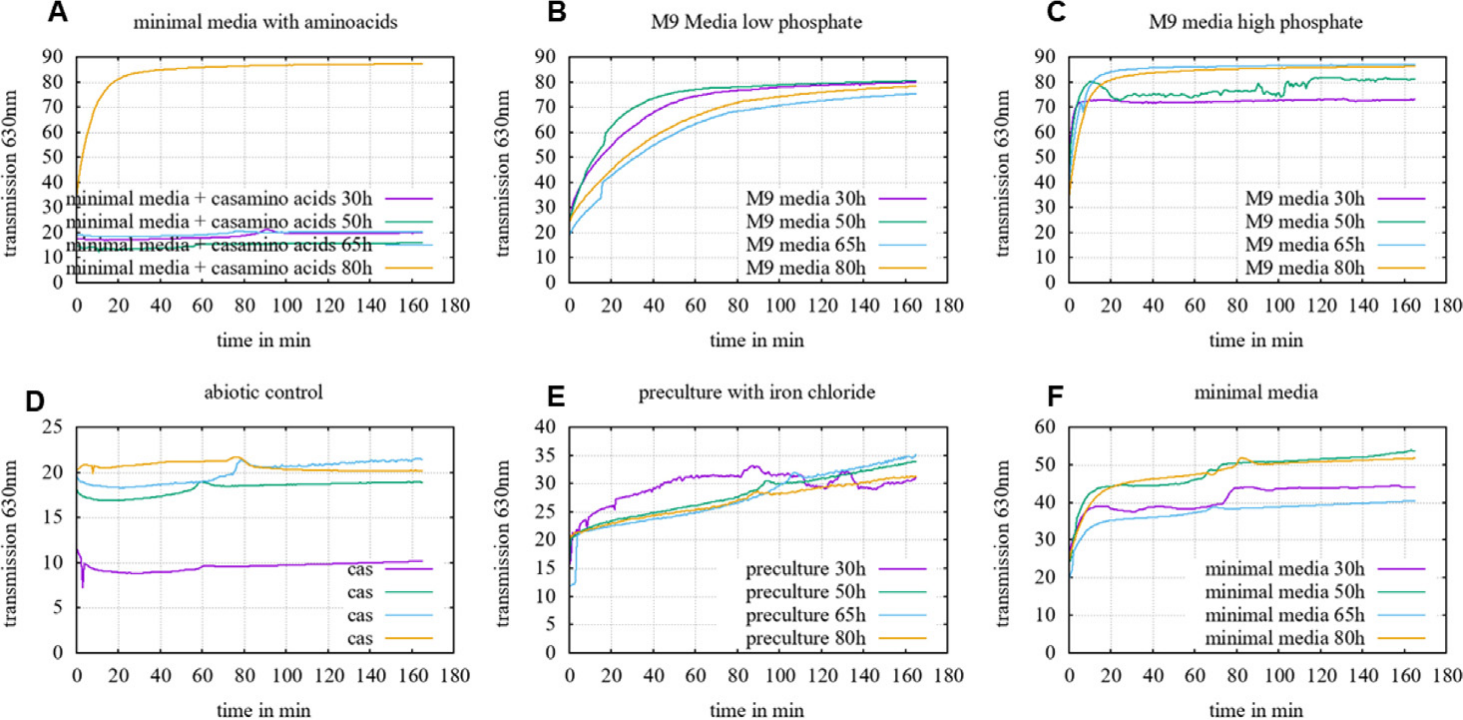
**CAS activity in dependency of time to investigate the optimal time point for siderophore detection and evaluation of the method in the different media.***A: minimal medium with casamino acids, B: M9 medium with low phosphate content (1.3 g L^−1^), C: M9 medium with high phosphate content (12.8 g L^−1^), D: abiotic control of M9 medium (Bosello et al., 2013), E: negative control preculture with iron chloride, F: minimal medium (basal salt solution as described in (Bosello et al., 2013), selected M9 medium and the LB preculture were analysed by time resolved measurements of the transmission during the first 3 hours after sampling to record the velocity of the decreased blue colour at 630 nm over time by an microplate reader with a reading rate from 1 read at per 4 sec.*

A siderophore relevant gene cluster was determined and therefore the production of siderophores by strain CWB2 was investigated. Therefore, we used a modifed CAS-assay [1]. To optimize the timescale for measurements a time-resolved decolorisation of chromazurol S (CAS) was recorded. This time-resolved CAS profile analysis at 30, 50, 65 and 80 h in dependency of different medium compositions showed highest siderophore production in M9 medium [3] with decoloration rates > 80% after 65 h (Fig. 1).

**Fig. 2:**
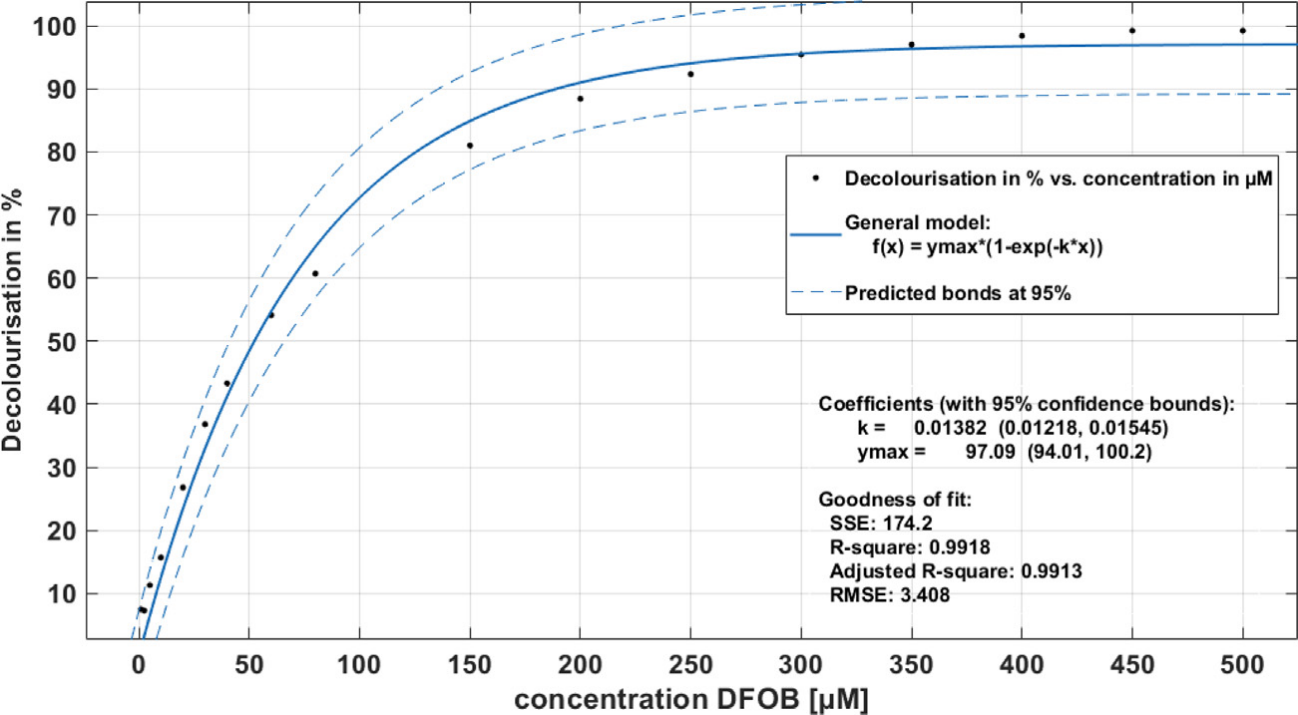
**DFOB calibration***curve derived by the liquid CAS assay, range from 10 – 500 µM measured in a 96 well plate in a solution of 1:1 medium: analysed after 4 h reaction time at 630 nm. The percentage CAS equivalents were then calculated with the formula given in the methods section DFOB CAS calibration described by Schwabe et al. (2019) into DFOB equivalents**[1]*.

Based on the evaluation presented in Fig. 1 we had selected a constant time point (after 4 h) for measurement of the percentage CAS-response. It was calculated on the difference of the absorbance of the full coloured blue Fe-CAS complex and a decolorized (yellow to reddish) sample at 630 nm. In dependency of the concentration the sample was diluted with M9 medium which served also as a blank. Desferrioxamine B (DFOB) served as a positive reference as it was commercially available as a pure siderophore and could be diluted in M9 medium in different concentrations to calibrate the CAS decolourisation to µM DFOB equivalents in the range of 1-500 µM. Data were fitted to an exponential decolourisation model function to obtain µM DFOB equivalents in µM (Fig. 2).

**Fig. 3:**
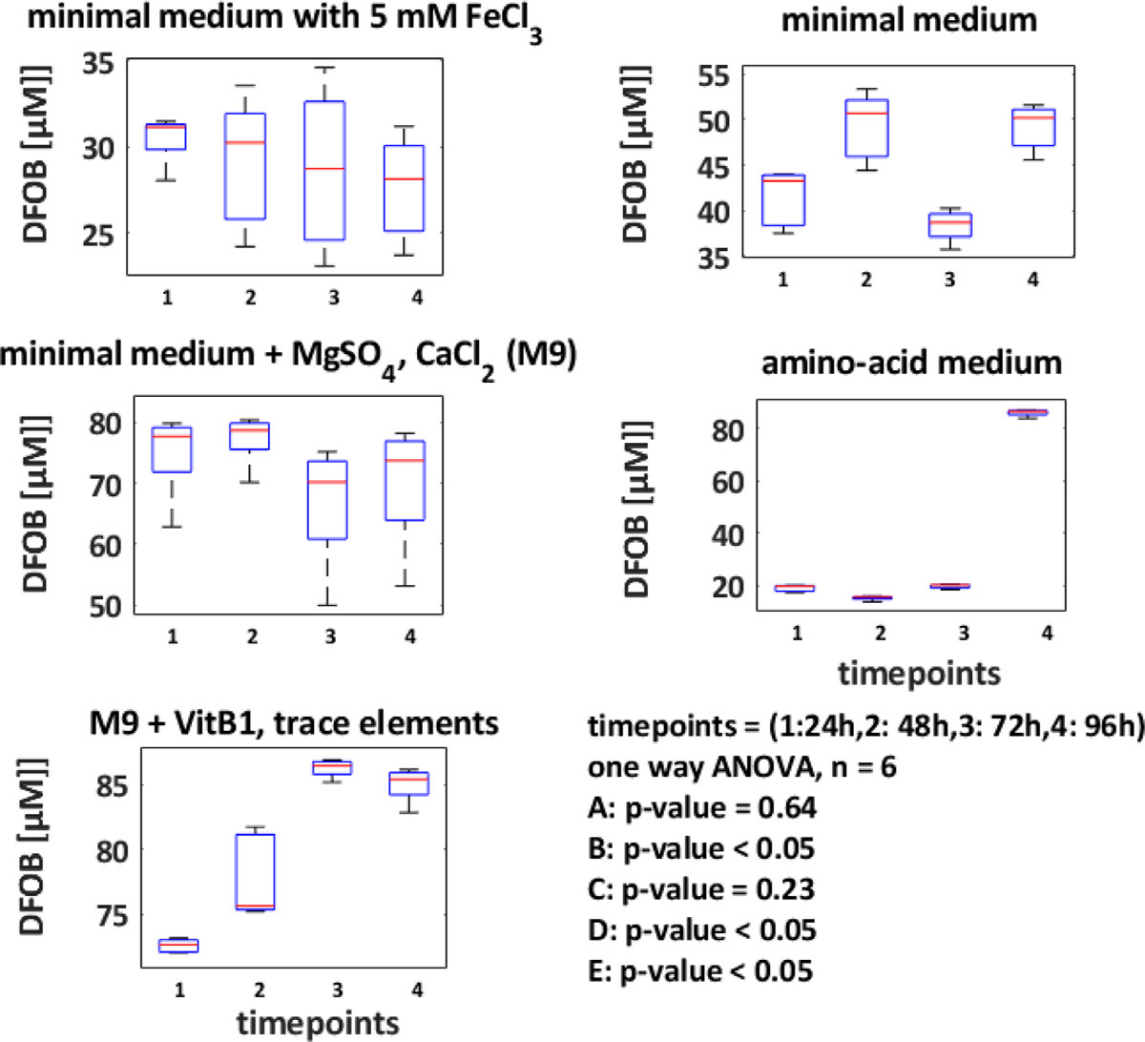
**Siderophore production detected by the liquid CAS assay for different medium compositions.***Here 15 ml falcon tubes were used and filled up wih 7 ml of medium and inoculated with a M9 preculture of Gordonia rubripertincta CWB2 with an initial OD_595_ of 0.05. Four time points are taken with 6 biological replicates. Afterwards variance of means was compared by ANOVA. As carbon source 4 g L^−1^ glucose were choosen. A: reference with FeCl_3_, B: medium with casamino acids, C: minimal medium basal salt solution, D: C with MgSO_4_ and CaCl_2_ (M9 medium), E: M9 medium as used in (Schwabe et al., 2018)**[4]*.

For optimisation of the siderophore production in dependency of medium composition CAS activities were measured and calculated to µM DFOB equivalents. Screening was done in 15 ml falcon tubes. Biological triplicates were measured at four time points. In Fig. 3 time point grouped boxplots of 4 different medium compositions are shown in which we applied an amino acid based medium (minimal medium supplemented with 4 g L^−1^ casamino acids) a minimal medium supplemented with MgSO_4_ and CaCl_2_, the M9 medium [3] and finally a minimal medium supplemented with 5 mM FeCl_3_ as a negative control with inhibited siderophore production. ANOVA analysis of the data showed that the M9 medium supplemented with vitamin B1 (thiamine; VitB1) and trace elements resulted in the highest DFOB CAS equivalents (85 µM) after 72 h cultivation, measured after 120 min CAS reaction time (see Fig. 3).

Further optimisation of siderophore production was done in 1 L scale (biological triplicates) in the M9 medium in dependence of different concentrations of succinate. Succinate presented the most promising carbon source (Table 2). OD measurement and CAS assays were performed to estimate DFOB production. Herein we observed 291 µM DFOB equivalents when *Gordonia rubripertincta* CWB2 was cultivated with 8 g L^−1^ disodium succinate. The yield of the XAD crude extract was 121 mg L^−1^.

**Table 2:** **Extraction yields of siderophores.** The XAD extractions were obtained from 3 different cultivations of 1 L each with different succinate concentrations during the cultivation period of 158 hours at 30°C in M9 medium given in mM DFOB equivalents derived by CAS activity measurements and mg L^−1^.

Production step	Units	Succinate 4 g L^−1^	Succinate 8 g L^−1^	Succinate 12 g L^−1^
supernatant 158 h	DFOB CAS equivalent [µM]	336 ± 1	291 ± 21	282 ± 11
flowthrough	DFOB CAS equivalent [µM]	18 ± 1	142 ± 6	126 ± 15
methanolic XAD extract	DFOB CAS equivalent [µM]	1291 ± 32	1547 ± 19	1029 ± 35
volume = 300µl µM CAS DFOB-eq.	[mg L^−1^]	216	249	172
Practical yield per liter [mg L^−1^]	[mg L^−1^]	143 ± 25	121 ± 35	178 ± 32

After having a robust production system for siderophores, the respective purification was established. Different methods of siderophore purification were applied. Collected fractions of HPLC and FPLC were analyzed by means of LC-MS. Therefore, 3 × 1 L cultures were extracted with XAD and eluted with 30 ml methanol, each. Extracts were further purified by C18-chromatography, like described in the methods section or by means of FPLC purification [5]. Samples derived of these procedures were analyzed by LC-MS/MS. Table 3 shows the comparison of 3 different purification strategies, which show the increased precursor intensity of fraction 12 after C_18_-chromatopgraphy. FPLC purification can also be combined with desalting without significant loss of XAD crude extract contents, but without the need of further concentration steps. Lyophilization resulted in degradation of the Ferrioxamine E.

**Table 3:** **Compounds detected in LC-MS/MS positive mode for 3 different purification strategies of siderophores from strain CWB2.** The sum of the precursor intensity related to the number of observed parent mass counts for the siderophore extracts from *Gordonia rubripertincta* CWB2. As a reference the iron complexed desferrioxamine B was used in the LC-MS workflow. Replicates of samples were averaged and compared for FPLC purified, HPLC and C_18_ concentrated, raw XAD and lyophilized crude extract were compared

Ferrioxamine E [M+Fe-2H]	Sum precursor Int	Theoretical Mass [M+Fe-2H]	Measured Mass [M+Fe-2H]	Δppm
HPLC fraction 12 purified C18 concentrate	128759887	653.259	654.572	1.22
FPLC fraction 44	40874925	653.259	654.576	1.07
raw XAD extract 10 mg /ml in MeOH	36960004	653.259	654.582	1.08
Lyophilisate10 mg/ml in MeOH	24817869	653.259	654.575	1.07

**Fig. 4:**
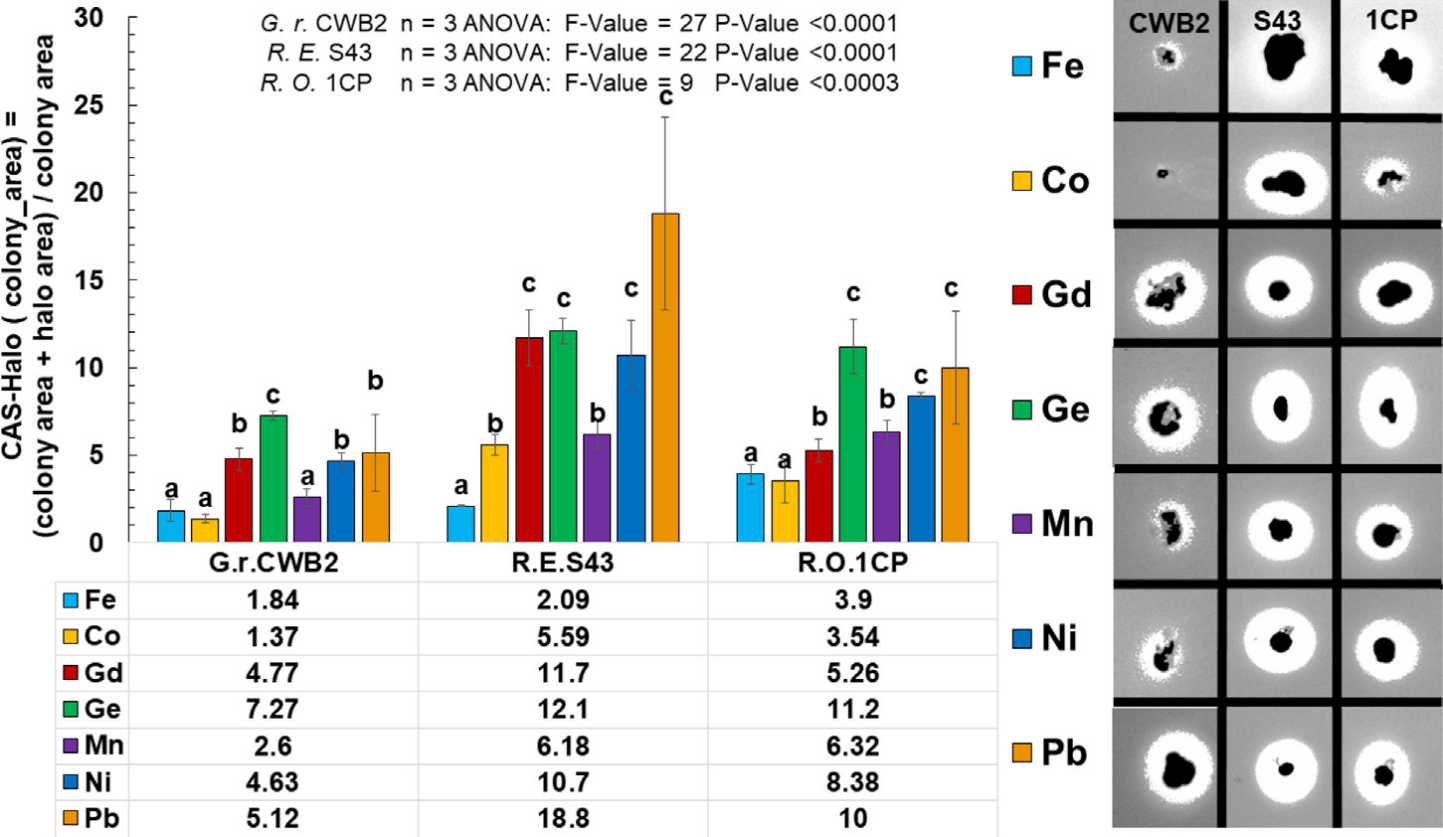
**Solid CAS assay combined with metal stress for *Gordonia rubripertincta* CWB2, *Rhodococcus erythropolis* S43 and *Rhodococcus opacus* 1CP**. *Bar plot visualization of the image analysis from the solid CAS plates combined with Co, Fe, Gd, Ge, Mn, Ni and Pb as a combined grouped scatter bar plot. Significant differences between means are identified by ANOVA and fishers LSD test. Means with same letters are not statistically significant at α = 0.05.*

Stress often induces the production of secondary metabolites and was therefore investigated. Siderophore production under metal stress was studied for *Gordonia rubripertincta* CWB2 and *Rhodococcus opacus* 1CP on Fe-CAS agar plates supplemented with different metals (final conc. 2 mM). The amount of siderophores produced and secreted by a bacterium can be measured on such agar plates on base of halo formation. Therefore, siderophore halo formation was normalized on colony area and were calculated from image analysis derived pixel-areas. Pictures were taken after 6 weeks and were cropped interactively (Fig. 4) for each plate and colony replicate (3) to create a cropping list. After cropping of the colony positions, double thresholding was performed interactively (Fig. 4), firstly for the colonies and secondly for the siderophore produced halos around the colonies. Sum of filtered colony and halo-pixels were divided by the sum of filtered colony-pixels. The given ratio was plotted here in bar plots grouped by strain and metal, which was used to induce metal stress to investigate conditions, which trigger siderophore production. The herein observed results indicated siderophore triggering by Gd and Ge for strain CWB2 and by Ge, Ni and Pb for *Rhodococcus opacus* 1CP. The latter strain was used as comparative actinobacterium supposed to produce heterobactin siderophores. In addition, *R. erythroplois* S43 was used which was recently shown to produce heterobactin like structures upon As stress [6].

**Fig. 5:**
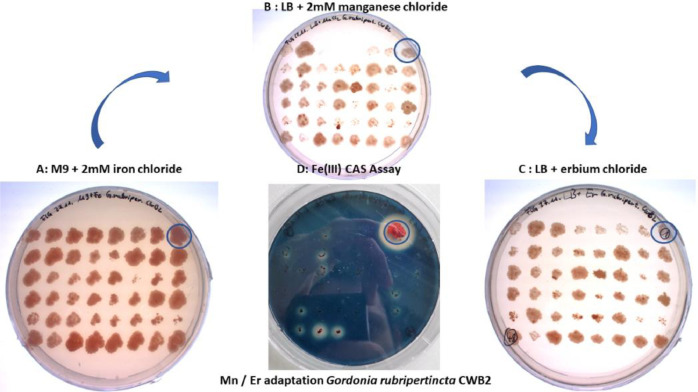
**Adaptation of growth and siderophore production of *Gordonia rubripertincta* CWB2 during supplementation with MnCl_2_ and ErCl_3_.***A: Cultivation of a MnCl_2_ cultivated preculture, which was separated on MnCl_2_ agar plates and picked to a freshly prepared M9 agar plate which contained 2mM FeCl_3_. B: Transferred colonies from a plate (from A) cultivated under 2mM MnCl_2_ concentration. C: MnCl_2_ cultered cells from plate B were cultivated on a freshly prepared 2 mM ErCl_3_ agar plate. D: Fastest grown colony “1” marked with a blue circle was observed to produce most siderophores.*

In previous CAS assays the influence of manganese on CAS activity was not significant but a decreasing of growth in LB medium supplemented with MnCl_2_ was observed. *Gordonia rubripertincta* CWB2 showed only poor growth on CAS agar plates, with only one colony grown after 6 weeks at RT. Therefore, we applied an adaptation procedure to manganese and erbium on LB and M9 agar plates. The colony, which showed highest growth and CAS activity (Fig. 5) was separated and used for further experiments.

**Fig. 6:**
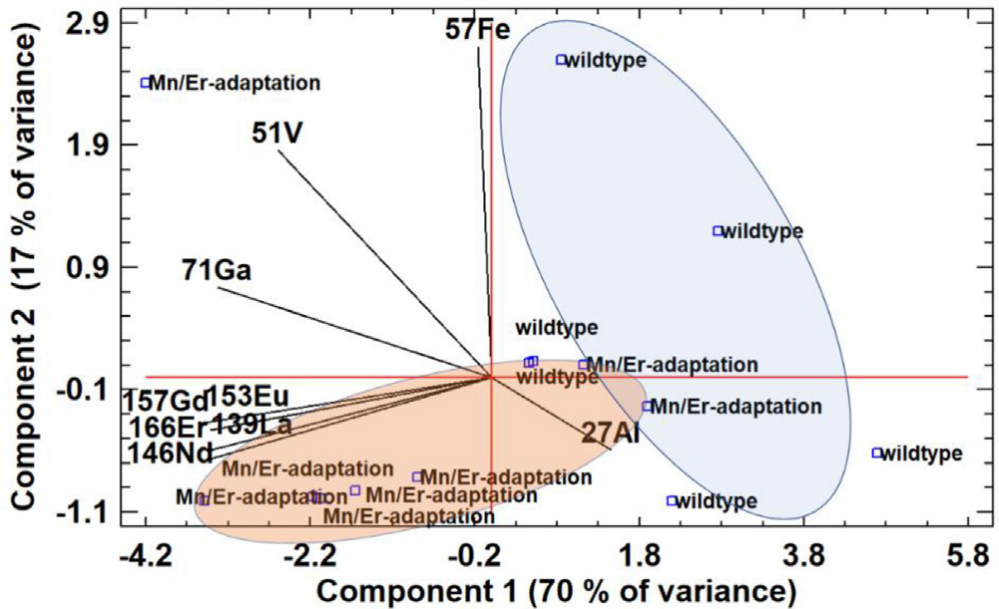
**PCA analysis of cell dry mass biosorption of Fe, Al, Ga, V, and the rare earth's La, Nd, Eu, Gd and Er by the *Gordonia rubripertincta* CWB2 and its Mn / Er adapted variants**. *Principal component analysis shows a clustering of the biological treatments on biosorption of the different metals. Gordonia rubripertincta CWB2 cells cultivated under wild-type conditions showed a preference for iron (blue colored region), whereas the under Mn/ Er adaptation derived cells clearly showed an increased biosorption of the rare earths La, Nd, Gd, Eu and Er (red colored region). Cells were cultivated in LB-medium, harvested by centrifugation and lyophilized to dryness.100 mg dry cell biomass was mixed with 10 ml 2 mM element standard solution pH 2 and shaken for 1 h. Cells were centrifugated and the supernatant was analysed by ICP-MS.*

16S-rRNA gene sequencing of the V3-V4 region of the wild-type strain and their Mn/Er adaptation showed pure DNA of *Gordonia rubripertincta* CWB2 (data not shown). PCA analysis of the metal biosorption of the wild-type and the Er/Mn adaptation from an acidic multi-element solution in dependency of the elements Fe, Ga, V, Al, La Nd, Eu, Gd and Er, demonstrated clearly a significant effect (see Fig. 6 and Table 4). For the Er/Mn adaptation *Gordonia rubripertincta* CWB2 increased metal concentrations of rare earth's La, Nd, Eu, Gd were measured in the eluates in comparison to the wild-type *Gordonia rubripertincta* CWB2.

**Table 4:** **ANOVA analysis of the target elements concentration in supernatants** of multi-element solutions containing Fe, Al, Ga, V, and the rare earth's La, Nd, Eu, Gd and Er. Biosorption of 100 mg dry biomass of *Gordonia rubripertincta* CWB2 and its Er/Mn adapted variants were investigated (see Fig. 6). Significant differences between means are identified by ANOVA and fishers LSD test. Means with same letters are not statistically significant at α = 0.05.

element	Al in µg / ml	Fe in µg / ml	Ga in µg / ml	La in µg / ml	Nd in µg / ml	Gd in µg / ml	Eu in µg / ml	Er in µg / ml
Multi-element solution	1.67a	0.99a	1.67a	2.20a	2.34a	2.41a	2.44a	2.52a
ErCl_3_/MnCl_2_ adaptation	1.82 ± 0.5a	3.32 ± 1.12b	0.52 ± 0.17b	1.11 ± 0.36b	1.11 ± 0.34b	1.25 ± 0.34b	1.22 ± 0.34b	1.13 ± 0.3b
Wild-type	1.93 ± 0.49a	3.66 ± 0.74b	0.34 ± 0.12c	0.58 ± 0.23c	0.6 ± 0.24c	0.72 ± 0.31c	0.69 ± 0.29c	0.65 ± 0.2c
P-Value	0.7	0.55	0.0572	0.01	0.01	0.01	0.01	0.01
F-Ratio	0.15	0.3	4.62	9.2	9.1	8.4	8.1	8.5

**Fig. 7:**
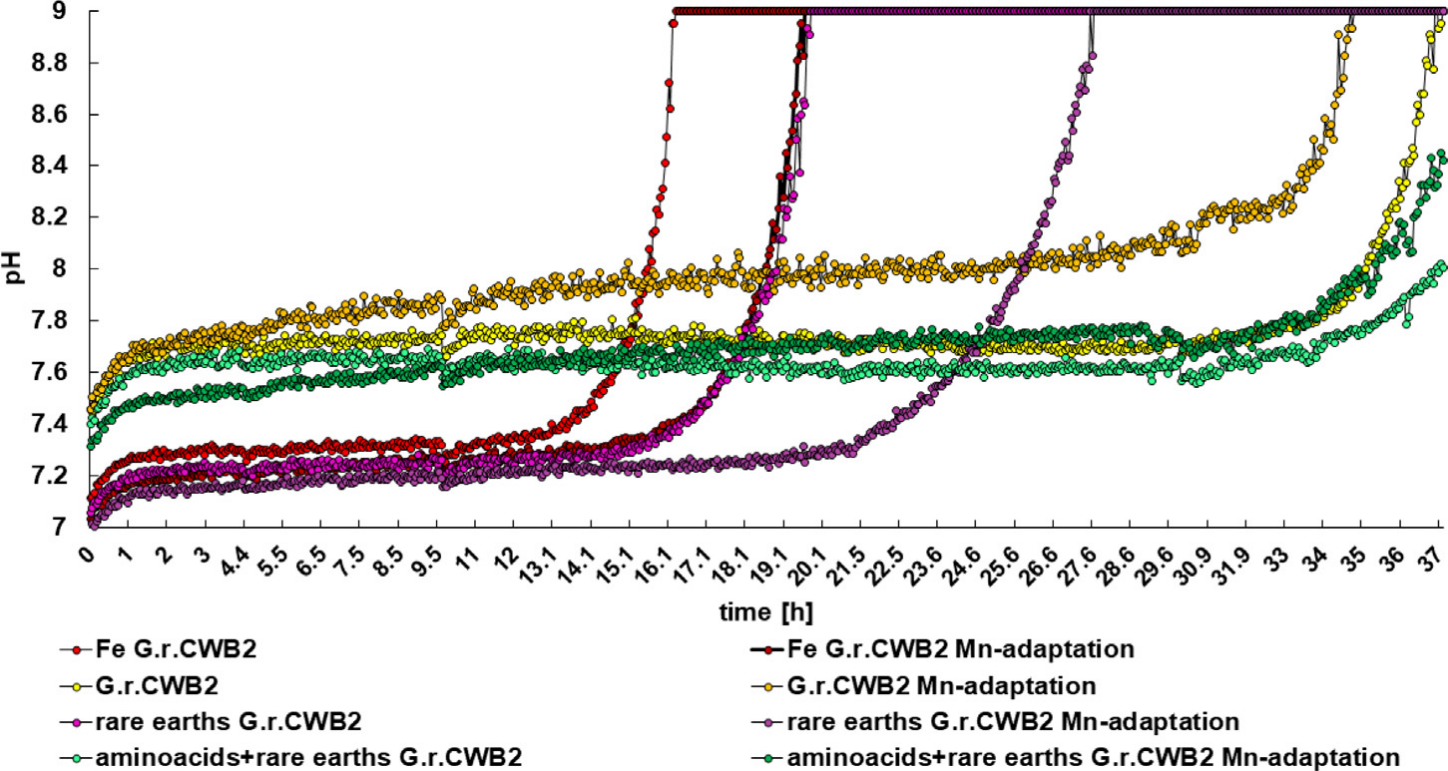
**Cultivation-dependent pH profiles of *Gordonia rubripertincta* CWB2 and its Er/Mn adaptation in dependency of supplementation with 5 rare earth elements (2 mM La, 2 mM Nd, 2mM Gd, 2 mM Eu, 2 mM Er).***Rare earths and additionally casamino acids (4 g L^−1^) were applied for cultivation. As a negative control for siderophore production samples were supplemented with 5 mM FeCl_3_.*

The pH in medium variations for cultures of *Gordonia rubripertincta* CWB2 and their selected adaptations were measured during cultivation in M9 medium in dependency of rare earth's (2 mM La, 2 mM Nd, 2mM Gd, 2 mM Eu, 2 mM Er), casamino acids and iron limitation to relate siderophore production to pH values (Fig. 7). All cultures increased the pH in the media up to pH 9. For cultivation with rare earths and amino acids the changes in pH were observed after 72 h and do not differ significantly between the 2 variants of *Gordonia rubripertincta* CWB2. The reference groups without additional metal stress showed that the adaptation reached earlier higher pH values than the wild type. Including the supplementation with 2 mM rare earths during cultivation the time scale difference reached the maximum. The wild type *Gordonia* reached the pH maximum pH 9 after 15 h, the adaptation after 19 h. The same effect was observed for the control groups (M9 medium supplemented with iron).

**Fig. 8:**
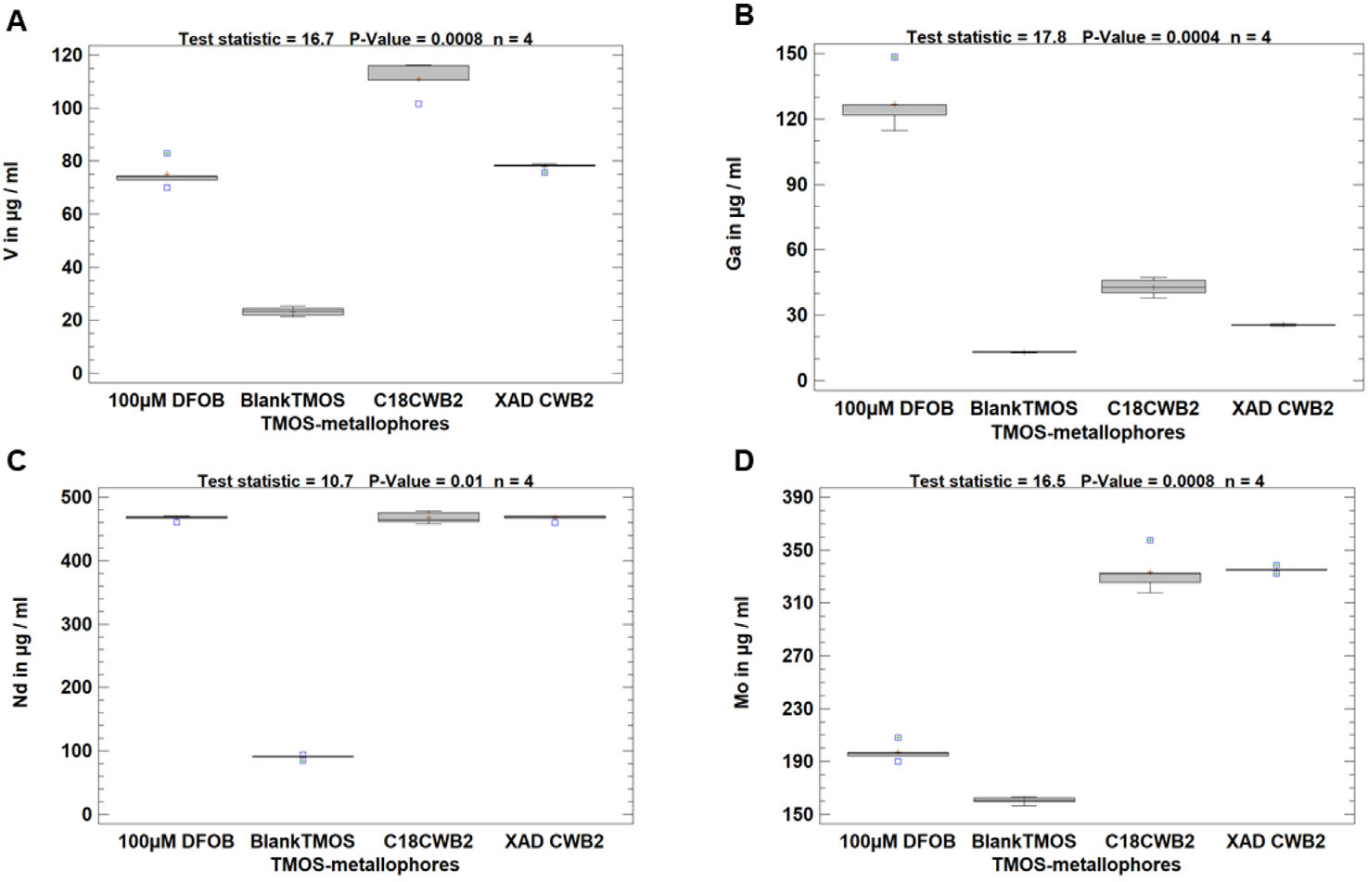
**TMOS entrapment as a metal immobilizing screening***for metallophore crude extracts used in a metal desorption study involving batch adsorption of metals onto metallophore functionalized TMOS powders from XAD crude extracts of Gordonia rubripertincta CWB2. Box plots of 4 replicates were visualized and shown for Ga, V, Nd, and Mo concentrations in µg/ml eluate (500 mM EDTA/ DTPA). Significant differences between means are identified by ANOVA and fishers LSD test. Means with same letters are not statistically significant at α = 0.05.*

Besides siderophore production we investigated their applicability. Here the main issue is stabilization and reuse. Therefore, the immobilization of siderophore samples was studied. Metallophore containing XAD extracts were entrapped in tetramethoxysilane (TMOS) and grounded to powder. Metal adsorption/desorption on the functionalized carrier was performed in a model solution, which contained V (931.1 ± 74.8 µg mL^−1^), Ga (76.4 ± 7.4 µg mL^−1^), Mo (1123 ± 86.7µg mL^−1^), and Nd (558.3 ± 113.7 µg mL^−1^). After shaking of 20 ml with 1 g functionalized carrier the samples were centrifuged, and the metals adsorbed onto the carrier pellet were eluted with 500 mM EDTA/DTPA solution. The element concentration in the eluate was measured by ICP-MS. With the 100 µM DFOB-TMOS a significant effect for Ga (126.7 ± 12.9 µg mL^−1^) with a concentration factor of about 10 in comparison to the reference blank carrier material (13.1 ± 0.3 µg mL^−1^) was observed. For vanadium, the highest concentration factor was observed for the C_18_–derived concentrate (see above) from *Gordonia rubripertincta* CWB2. For neodymium, all metallophore-TMOS materials show significant increases. For molybdenum especially the extracts of *Gordonia rubripertincta* CWB2 show significant enrichment of the target metal in comparison to the reference with about 2-fold increase (see Fig. 8 and Table 5).

**Table 5:** **Metal concentrations of EDTA/DTPA eluates from metallophore functionalized TMOS powders from *Gordonia rubripertincta* CWB2 after XAD extraction and after C_18_-chromatography.** Significant differences between means are identified by ANOVA. Means with same letters are not statistically significant at α = 0.05.

TMOS-metallophores	V in µg / ml eluate	Ga in µg / ml eluate	Mo in µg / ml eluate	Nd in µg / ml eluate
BlankTMOS	23.3 ± 1.7a	13.1 ± 0.3a	160.4 ± 2.6a	90.3 ± 3.3a
DFOB	74.9 ± 4.8b	126.7 ± 12.9b	197.1 ± 6.6a	466.9 ± 3.9b
C_18_ CWB2	111 ± 6c	42.8 ± 3.9c	333 ± 15b	467.6 ± 8.6b
XAD CWB2	78 ± 1.3b	25.5 ± 0.4c	335.2 ± 2.3b	467.4 ± 4.3b
test statistic	16	17	16	10
ANOVA P-value	0.0008	0.004	0.008	0.013
µg/ml elements in HNO_3_ leachate pH 4	931.1 ± 74.8	76.4 ± 7.4	1123 ± 86.7	558.3 ± 113.7

**Fig. 9:**
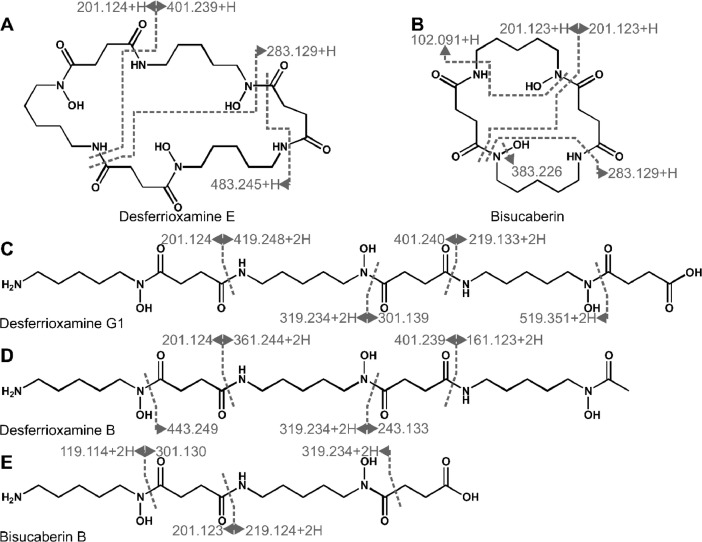
**Fragment annotation of molecules with siderophore function, detected in LC-MS/MS-ESI^+^.***Fragmentation sites are indicated by dark grey lines and corresponding fragments are labeled by the detected masses. Given are significant figures. Annotation of fragment spectra was aided by MetFrag**[10]*.

**Fig. 10:**
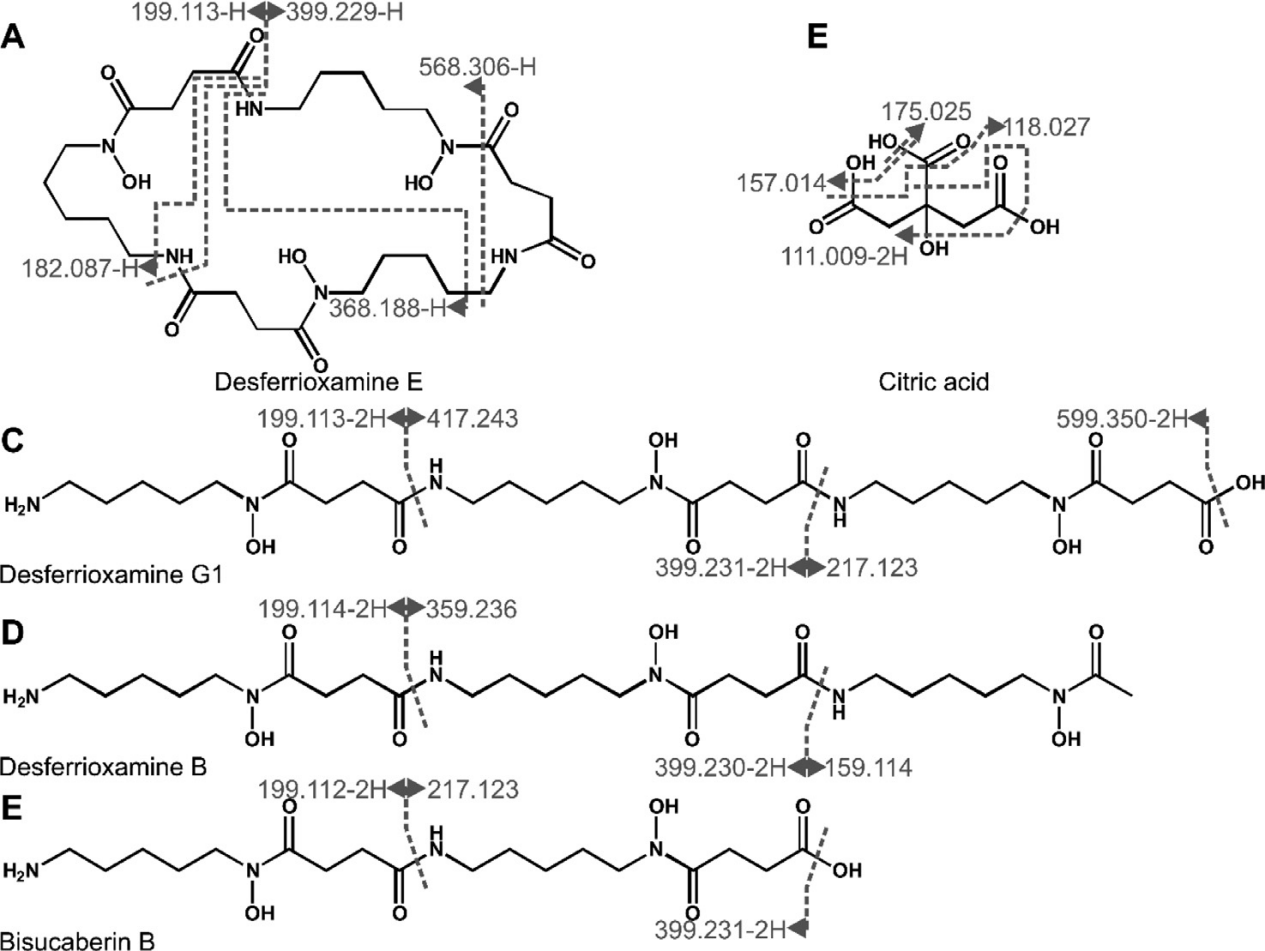
**Fragment annotation of molecules with siderophore function, detected in LC-MS/MS-ESI^−^.***Fragmentation sites are indicated by dark grey lines and corresponding fragments are labeled by the detected masses. Given are significant figures. Light grey lines do not display fragmentation sites but contiguous fragments originating from fragmentation at multiple sites. Annotation of fragment spectra was aided by MetFrag**[10]*.

Siderophores from XAD extracts were enriched by HPLC fractionation and identified by LC-MS/MS measurements in positive and negative mode combined with molecular networking, using the global natural products social molecular networking (GNPS [7]) platform (Fig. 9, Fig. 10; Tables 6 and 7). The methods were adapted from previous works [8,9]. Parent masses and fragment spectra were compared to online libraries (GNPS [7], MetFusion), to identify desferrioxamine and bisucaberin siderophores as well as citrate. Through molecular networking molecules were identified with fragmentation patterns similar to the desferrioxamines, which are likely to be derivatives and analogues [9]. Overall, 28 desferrioxamine-like molecules were found in positive mode and 14 in negative mode. Annotation of fragment spectra onto molecular structures was guided by *in silico* fragmentation, using MetFrag [10].

**Table 6:** Compounds detected in LC-MS/MS positive mode.

Name	measured mass [M+H]^+^	theoretical mass [M+H]^+^	Δppm	HPLC fraction
Desferrioxamine G1 (Barona-Gómez et al., 2004)	619.3629	619.3667	6.082	14
Desferrioxamine E (Barona-Gómez et al., 2004)	601.3571	601.3561	1.660	10, 14, 15
Desferrioxamine B (Barona-Gómez et al., 2004)	561.3583	561.3612	5.145	15
Bisucaberin B (Fujita et al., 2013)	419.2486	419.2506	4.711	14, 15
Bisucaberin (Kadi et al., 2008)	401.2374	401.2400	6.505	10, 14, 15
	633.3788			14
	613.3549			10
	584.3267			10
	547.2941			10
	515.2682			10
	501.2523			10
	403.2511			10
	400.2031			15
	348.1735			10
	317.1563			10, 14, 15
	315.1511			10, 15
	310.1809			14, 15
	302.2031			10
	299.6374			14
	281.1667			15
	260.1752			10, 14, 15
	257.1451			10
	252.9893			10, 14
	246.1288			5, 14
	233.1450			10, 14, 15
	219.1318			10, 14, 15
	217.1503			10
	203.1350			10

**Table 7:** Compounds detected in LC-MS/MS negative mode.

Name	measured mass [M-H]^−^	theoretical mass [M-H]^−^	Δppm	HPLC fraction
Desferrioxamine G1| (Barona-Gómez et al., 2004)	617.3519	617.351017	1.430	14, 15
Desferrioxamine E (Barona-Gómez et al., 2004)	599.3416	599.340452	1.915	10, 14, 15
Desferrioxamine B (Barona-Gómez et al., 2004)	559.3461	559.345538	1.005	15
Bisucaberin B (Fujita et al., 2013)	417.2413	417.234925	5.093	10, 14, 15
Citric acid (Adam et al., 2015)	191.0354	191.019177	5.879	5, 26
	631.3751			14
	614.3494			10
	582.3220			10
	568.3063			10
	543.3589			14
	517.3422			10, 14, 15
	503.3268			10
	430.2234			10
	401.2461			10, 14
	400.2134			10

Only 5 of the siderophore-like molecules were identified by data mining and comparing with library entries. The others, here with fragmentation spectra similar to desferrioxamines, remain to be characterized, regarding their structure and differences in metal affinity.

**Fig. 11:**
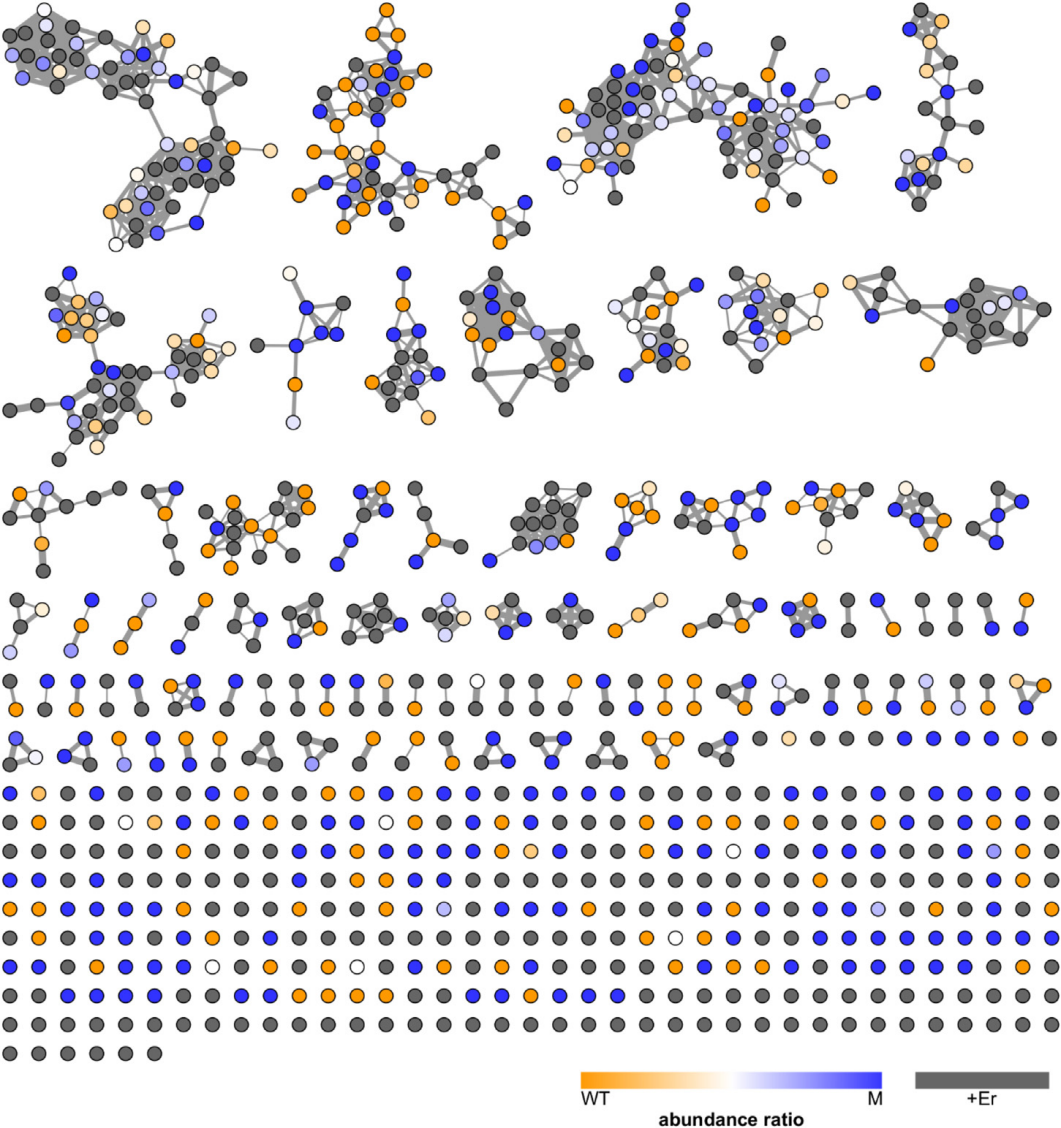
**Molecular network of the secreted metabolome of *G. rubripertincta* CWB2 (WT) and an Mn/Er preadapted variant (M).***Nodes represent metabolites as detected in culture supernatants by LC-MS/MS-ESI^+^. Node colours indicate whether a metabolite was more abundant in wild type cultures (orange), in the preadapted culture (blue), or equally abundant (white). Grey colour indicates that a molecule was only found after erbium was added to either one or both cultures. Nodes are connected if the cosine similarity of corresponding fragment spectra is ≥ 0.7 and the thickness of connecting lines indicates similarity.*

**Fig. 12:**
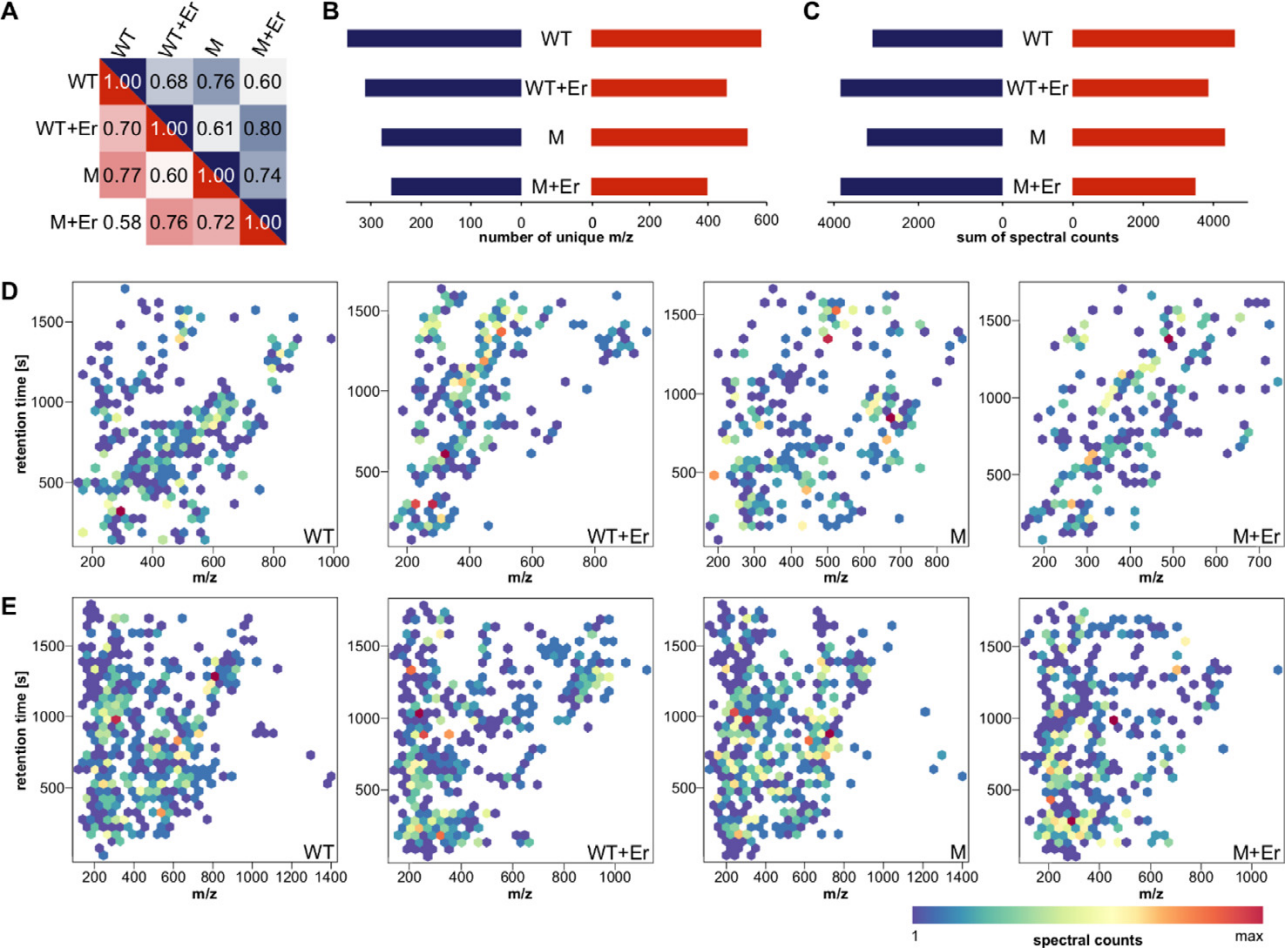
**Comparison of mass spectrometric profiles. A***Similarity of secreted metabolomes. Cosine similarity scoring based on all signals observed in* liquid chromatography-coupled *mass spectrometry. Colour code: positive mode – blue; negative mode – red.****B****Chemical diversity based on the number of unique signals observed in mass spectrometry.****C****Amounts of compounds based on the sum of spectral counts observed in mass spectrometry.****D****Secreted metabol*ite *profiles of positive mode data. Maximum counts: WT – 130; WT+Er – 127; M – 98; M+Er – 161.****E****Secreted metab*olite *profiles of negative mode data. Maximum counts: WT – 107; WT+Er – 99; M – 84; M+Er – 84.*

We investigated the secreted metabolomes of wild-type *Gordonia rubripertincta* CWB2 (WT) and erbium/manganese preadapted *Gordonia rubripertincta* CWB2 (M) (Fig. 11, Fig. 12). Even if no rare earths such as erbium were added to the cultivation media, the secreted metabolome of the wild-type diverged from the adapted variant (Fig. 11).

The addition of 2 mM erbium chloride strongly impacted the secreted metabolome of wild-type *Gordonia rubripertincta* CWB2 (WT) as well as the adapted variant (M) (Fig. 12). Comparing overall metabolomic profiles (as presented in Fig. 11) of positive and negative mode measurements by cosine similarity, profiles of WT against M+Er and WT+Er against M show the greatest differences. WT+Er compared to M+Er and WT against M are the most similar ones (Fig. 12A). Both strains produced a similar diversity of compounds under +/- Er-conditions (Fig. 12B), while the addition of erbium seems to induce the production of more compounds measurable in positive mode, and less compounds in negative mode (Fig. 12C). Overall the preadaptation to Er/Mn as well as the addition of 2 mM erbium chloride to the cultivation media affected the secreted metabolome, as for example the maximum m/z of secreted compounds was greater in the absence of erbium (Fig. 12D/E).

## 2 Experimental Design, Materials, and Methods

### 2.1 For genome mining

For genome mining we applied the tool antiSMASH 4 and 5 [2], for the investigation of the existing siderophore biosynthetic gene clusters in the genome sequence of *Gordonia rubripertincta* CWB2 (BioProject accession number: PRJNA394617; NCBI Reference Sequence: NZ_CP022580.1, [11]). The genome to product analysis tool MIBiG [12] was used to obtain a siderophore product prediction.

### 2.2 Production of siderophores from *Gordonia rubripertincta* CWB2

For production of siderophores from *Gordonia rubripertincta* CWB2 (DSM 46758) M9 minimal medium was used as described previously [4,11,13,14]. The minimal medium was supplemented with a trace element solution [3] and 4 g L^−1^ succinate as carbon source. A sub-cultivation was done with 3 g L^−1^ freshly grown LB biomass, which was resuspended in 100 mL iron-depleted medium and incubated for 55 h at 120 rpm and at 30°C. The sub-cultivated biomass was again harvested by centrifugation (yield of 4 g L^−1^) and finally transferred (0.4 g L^−1^*)* in new 3 L Erlenmeyer flasks comprising 1 L freshly prepared iron-depleted M9 minimal medium. For screening of siderophore production in dependence of different medium compositions, washed cell mass of strain CWB2 was inoculated at a final OD_595_ of 0.05 in 7 ml medium which contained the certain medium compounds, like the basal salt solution (further called minimal medium), the trace element solution and CaCl_2_, and MgSO_4_ (further called M9 medium). To optimize time scale for the CAS assay and its incubation time, kinetic time profiles were analysed for 4 h with a frequency rate of 1 scan per 4 sec.

### 2.3 Isolation of crude extracts by solid phase extracted with XAD

For isolation of the solid phase extracted XAD crude extracts 3 × 1 L of in M9 medium cultivated bacteria (0.3 g L^−1^ inoculum, 4 g up to 12 g succinate as a carbon source 158 h at 30°C) were used. Culture biomass was separated by centrifugation; a solid-phase extraction was applied to the supernatant. For this, a 10 g L^−1^ mixture of 5 g L^−1^ XAD4 and 5 g L^−1^ XAD16 were washed with water, methanol, and another time with water. The activated resin was added to the supernatant and shaken at 100 rpm at 4°C for 12 h. The supernatant was decanted, and the resin was washed with 1 L ddH_2_O L^−1^ medium. Elution of the siderophores was done with 100 mL methanol per 10 g of resin. The procedure was repeated three times to ensure optimal yield. The methanolic crude extract (2 L) was concentrated under reduced pressure (< 30°C) to a tenth of volume (200 mL). For further purification and application, the methanolic extracts were diluted with 100 ml H_2_O to separate the MeOH from the extracts in a rotary evaporator under reduced pressure until only the water remained.

### 2.4 Mn/Er adaptation of *Gordonia rubripertincta* CWB2

For Mn/Er adaptation of *Gordonia rubripertincta* CWB2 a freshly grown colony from a LB-agar plate was incubated at 30°C and shaken at 150 rpm overnight in 20 ml LB medium supplemented with 2 mM MnCl_2_. A 10^−6^-fold dilution of these precultures was than plated onto 10 LB-agar plates supplemented with 5 mM ErCl_2_. 48 selected colonies, which were observed earliest, were transferred into visual analysable raster positions on a new Er-LB agar plate. From that plate, grown colonies were transferred to a CAS agar plate as described earlier [1,4] (see Fig. 5) to select adapted variants with better growth and increased siderophore activity, further called Mn/Er adaptation of *Gordonia rubripertincta* CWB2.

### 2.5 Investigation of metal biosorption by *Gordonia rubripertincta* CWB2

For the investigation of metal biosorption by *Gordonia rubripertincta* CWB2 and Mn/Er adapted cells were cultivated in 250ml LB and M9 medium as described above, harvested by centrifugation and lyophilized. Cell pellets were weighed, aliquoted in 100 mg portions, and supplemented with 2 ml of a multi element solution containing 47 elements each at 2 mM. After vortexing the solution samples were shaken for 2 h at 30°C and 150 rpm. Cells were harvested by centrifugation. 100 mg of each sample was first mixed in a sample tube with 200 μl ultrapure water and 1.9 ml 65 % nitric acid. The samples were then mixed with 600 μl of a 4.8 % hydrofluoric acid and digested in the microwave (MLS-ETHOS plus) with a temperature program - heating to 200°C in 25 minutes, temperature maintained for 5 minutes, then continuously cooling to 75°C within 30 minutes. After heating, the samples were left to cool for about 2 hours. The completely dissolved samples were transferred into 15 ml sample tubes. Element concentrations in diluted samples (1:10) as well as solutions from the supernatant were measured by ICP-MS (xseries, Thermo Scientific) using 10 µg/l rhenium and rhodium as internal standard according to [15].

### 2.6 Measurement of time dependent pH profile by PresSens

For the investigation of differences in the pH profile of *Gordonia rubripertincta* CWB2 and its Er/Mn adapted variants we investigated growth during cultivation in a microplate system (24 wells) supplemented with a pH sensor (PreSens system by PreSens Precision Sensing GmbH).

### 2.7 Entrapment of siderophores in tetramethoxysilane (TMOS) sol gel beads

For embedding the siderophores in sol gel beads a nitric acid catalysed hydrolysis and condensation reaction of tetramethoxysilane (TMOS) and H_2_O is applied. For sol gel beads of 2 ml the reaction was set up in 24 well plates. A reactive mixture of 50 ml TMOS and 1.5 ml 10mM HNO_3_ (33.3:1 v/v) was stirred at room temperature (23°C) for 10 minutes in a 100 ml beaker. Afterwards 1.4 ml of this reactive mixture, 300 µl methanolic siderophore extract and 300 µl 100 mM phosphate buffer at pH 7 were mixed in each well. Further the plate was closed with an adhesive foil and pierced at each well with a pin to allow methanol fumes to vent. To reduce reaction speed temperature was set at 20°C or lower. Therefore, the plate was stored on ice in a polystyrene box for at least 5 days until the beads hardened. For further adsorption and desorption experiments of metals the sol gel beads were produced for each strain and beads without siderophores were produced as blank controls. Here data were collected and provided as supplemental material as supporting data sets (see suppl. material.).

### 2.8 Screening of metal adsorption/desorption studies batch experiments

For screening of metal adsorption/desorption batch experiments were performed in five replicates with the crushed immobilised metallophore powder. After 6 hours of shaking of 1 g carrier material, which refers to 10 mL of crude extract from 1 L of culture supernatant, at 120° rpm in 20 mL model solution, the metal analysis was carried out. The samples contained the following metals (see Table 8).

**Table 8:** Element concentration within the investigated model solution at pH 4.

V µg mL^−1^	Ga in µg mL^−1^	Mo µg mL^−1^	Nd µg mL^−1^
931.1 ± 74.8	76.4 ± 7.4	1123 ± 86.7	558.3 ± 113.7

The carrier material was separated by centrifugation and treated with 1 ml 500 mM EDTA to eluate the metals from the carrier. Each sample and a blank of the materials without biomolecules were acidified with 0.1 M HNO_3_ (10% v/v) filtered through a 0.22 µM filter-equipped syringe and prepared for ICP-MS measurement to obtain metal concentrations after the extraction with immobilized metallophores.

### 2.9 Modified CAS agar preparation and image analysis of CAS Halos

**Fig. 13:**
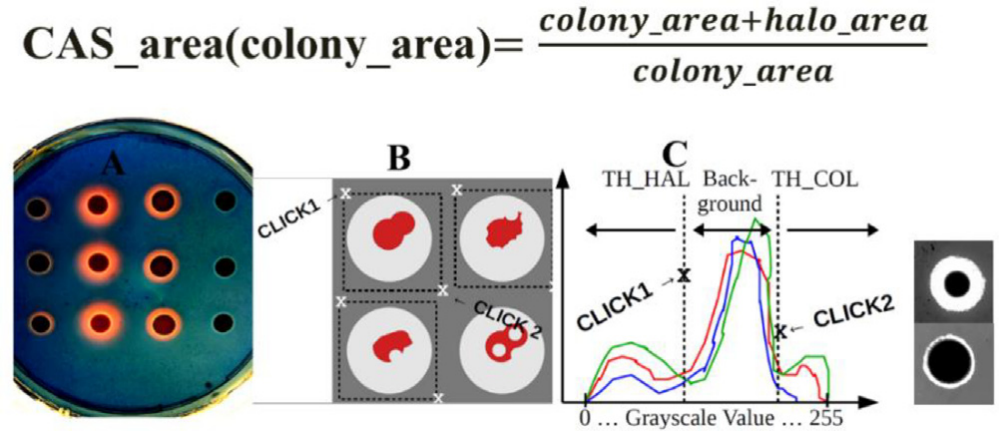
**CAS Agar Image screening procedure**. *A: Example of a CAS agar-plate containing the Fe-CAS blue coloured dye and 1 mM As_2_O_3_ as a stress trigger metalloid. B: Colonies were excised from the image with two clicks at the left upper corner and the right down colonies were cut out of the pictures by hand with sufficient distance to the halo with the help of two clicks left above the halos and right below the halos. C: To analyse the area of the halos and colonies, the threshold values of the halos and colonies were determined from each cut out under image of RGB pixel histograms in order to extract them from the background of the blue Fe-CAS dye.*

**Fig. 14:**
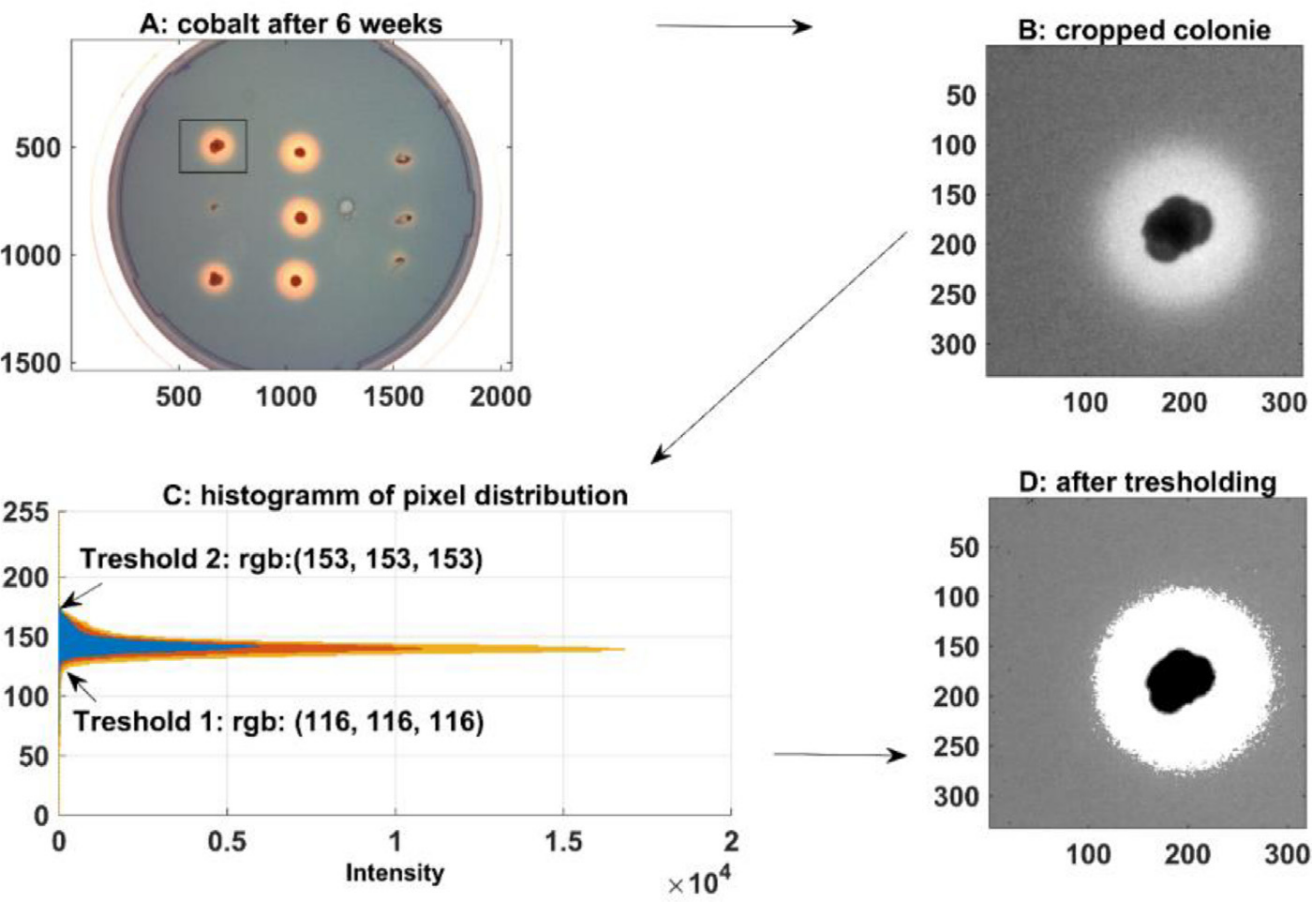
**Workflow example Image of *R. opacus* 1CP analysis of the solid CAS Assay.***A: Interactive cropping of the single colony; B: Cropped colony; C: Picking interactively the thresholds for colony and halo to remove the background; D Colonie (black: (0,0,0)) and halo (white (255,255,255)) after thresholding.*

We used the modified CAS agar procedure, a variant of the original solid CAS agar assay elsewhere [1,4] in order to screen for potential new compounds of metal stress resistant bacteria. In addition to iron, the metals Gd, Mn, Ger, Co, Ni, Pb, are used in final concentrations of 100 µM in order to analyze the resulting CAS agar halo. The resulting CAS performance was determined with standard image processing tools from pictures taken at the end state of experiments after 5 weeks. Specifically, the CAS_area in dependence of the colony_area was determined by all pixels of the halo_area nH and the colony_area related to the pixels of the colony_area (Figs. 13 and 14) and was used as an indicator for the performance of metal chelation under metal stress and iron limited conditions. The areas for halo and colony were determined using 2 interactively adjusted threshold operations on the input grayscale images.

Through visual analysis of the images, it was found that the background, the colony and the halo are located in different parts of the grayscale color range. The region of the colony is determined by all pixels with grayscale value smaller than tC, and the region of the halo is given by all pixels with grayscale value larger than tH. Firstly, the user is asked to crop the image into smaller parts containing single colonies, whereby the cropped image contains a border area with background. Second, for each one cropped sub image a histogram is plotted, which is used to determine the thresholds tC and tH. The procedure is repeated iteratively, and the output was verified visually. At the end of the procedure, the area of the halo and the colony regions are given by the sum of the pixels. We calculated from three colonies the average and the standard error. Significant differences between treatment groups were identified by the Kruskal Wallis test followed by a Dunn-Bonferroni post hoc method and marked with an asterisk (*), when they showed significant difference to the standard iron-CAS agar plate without additional metals (Fig. 4).

### 2.10 Identification of siderophores by LC-MS/MS

For structural analysis further purification steps as described in [1] were performed with C_18_-reversed-phase silica gel (particle size 15-25 µm, pore size 100 A, surface area 380 m^2^ g^−1^, fully end-capped, purchased from Merck #231-545-4), followed by HPLC fractionation and concentration under vacuum. To identify siderophores, HPLC fractions were subjected to LC-MS/MS measurements, using a nanoACQUITY-UPLC system (Waters) coupled to a Synapt G2-S HDMS (Waters) as described before [8]. In brief, molecules were separated on an AcquityUPLC HSS T3 column (Waters, pore size 100 Å, particle size 1.8 µm, inner cross section dimension 1 mm, length 100 mm), using a water acetonitrile gradient (Table 9) with 0.1% formic acid.

**Table 9:** Gradient used for LC-MS/MS analytics

Time [min]	% ACN with 0.1% FA
Initial	5.0
2.5	5.0
21.0	99.5
23.0	99.5
28.5	5.0
30.0	5.0

Molecules were selected for fragmentation through CID with argon in mass spectrometry, when the intensity exceeded 6000 counts/s. Data were evaluated as described earlier [8,9]. In brief, files were converted from waters raw to .mzXML using Proteowizard (ver. 3.0.9490) [16]. Subsequently, data were uploaded to the GNPS servers, the METABOLOMICS-SNETS workflow was applied [7], and visualized via Cytoscape (ver. 3.3.0). The annotation of fragment spectra was aided by MetFrag [10]. Overall this will help to identify more valuable metal chelating molecules useful in numerous future applications [17].

## Declaration of Competing Interest

The authors declare that there are no conflicts of interest.

## Appendix Supplementary materials

Image, application 1
